# Q-raKtion: A Semiautomated KNIME Workflow for Bioactivity Data Points Curation

**DOI:** 10.1021/acs.jcim.2c01199

**Published:** 2022-11-28

**Authors:** Deborah Palazzotti, Martina Fiorelli, Stefano Sabatini, Serena Massari, Maria Letizia Barreca, Andrea Astolfi

**Affiliations:** Department of Pharmaceutical Sciences, “Department of Excellence 2018-2022”, University of Perugia, Via del Liceo, 1, Perugia 06123, Italy

## Abstract

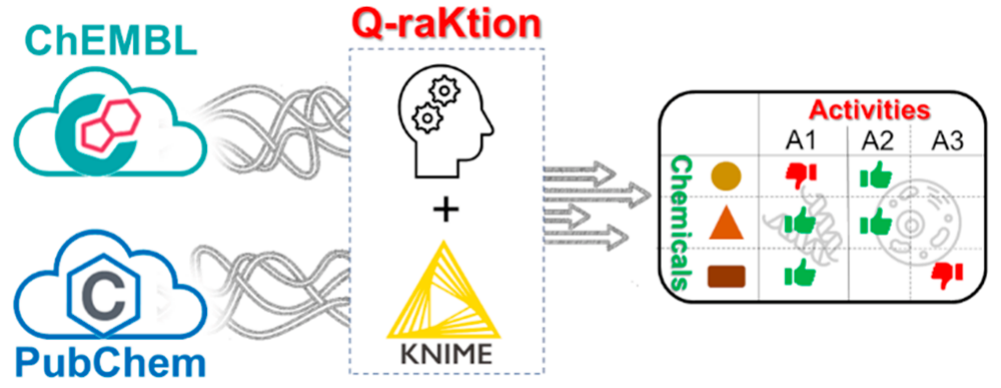

The recent increase of bioactivity data freely available to the scientific community and stored as activity data points in chemogenomic repositories provides a huge amount of ready-to-use information to support the development of predictive models. However, the benefits provided by the availability of such a vast amount of accessible information are strongly counteracted by the lack of uniformity and consistency of data from multiple sources, requiring a process of integration and harmonization. While different automated pipelines for processing and assessing chemical data have emerged in the last years, the curation of bioactivity data points is a less investigated topic, with useful concepts provided but no tangible tools available. In this context, the present work represents a first step toward the filling of this gap, by providing a tool to meet the needs of end-user in building proprietary high-quality data sets for further studies. Specifically, we herein describe Q-raKtion, a systematic, semiautomated, flexible, and, above all, customizable KNIME workflow that effectively aggregates information on biological activities of compounds retrieved by two of the most comprehensive and widely used repositories, PubChem and ChEMBL.

## Introduction

In the course of a traditional drug discovery project, a tremendous amount of data is produced relating to the chemical structure, biological activity, physicochemical properties, and many other characteristics of the compounds under study. Noteworthy, the massive information thus generated has triggered, especially in recent years, a growing trend toward data sharing and open data initiatives. Specifically, different types of data have been stored as data points on multiple publicly available informatic sources such as PubChem^1^ and ChEMBL.^2^ Therefore, access to these databases provides a valuable supplier of data for training predictive models using classical quantitative structure–activity relationship (QSAR) strategies or more advanced machine learning algorithms.^3^ However, the preparation of the initial data set requires deep attention and analysis, as a user interested in integrating data from different sources could be faced with two main issues.^4,5^

At first, each database has its own focus and objective that influence the type and degree of details of the data collected. In this regard, a recent analysis performed by Isigkeit and co-workers^6^ revealed considerable differences in terms of available compounds and associated bioactivities among five different chemical databases. Specifically, the authors found that less than 40% of the analyzed molecules were reported in more than one repository and that less than 1% of these compounds were shared among all of the repositories considered in the study. These results clearly highlight how the information relating to compounds/targets of interest is scattered over different sources, forcing the analysis of multiple databases to have an exhaustive data collection.^7,8^

Second, data coming from multiple sources may lack uniformity and coherence, and therefore the user should possess in-depth knowledge/expertise to integrate and harmonize the information appropriately to create high-quality data sets. In this context, the main obstacle that needs to be faced is the difference in the data points format and/or in the annotations used by the various databases.^5^ For example, by comparing different databases, the same compound might be labeled using dissimilar identifiers or the activity data points are reported in a nonhomogeneous way, as in some cases concentrations can be found (e.g., nM, μM), while in others the corresponding negative logarithm of the activity value is reported (e.g., pIC_50_ = −log_10_ IC_50_).

In this scenario, a careful inspection of the collected chemical and biological data is of fundamental importance, as was already highlighted in several studies.^4^ The term “data curation” therefore comes into play here, referring to an ensemble of activities aimed at the extraction, processing, and management of biological/chemical data used for the generation of a predictive model.

In the literature, different automated pipelines for the curation of chemical structures have emerged in recent years.^9−12^ Similarly, several examples have been reported describing the curation of bioactivity data points, generally aimed at creating comprehensive data sets of compounds that include data from multiple sources,^6,13−17^ by adopting ad hoc strategies for the purposes of specific projects. However, most of the published protocols are hard to read or reproduce for a noncomputational scientist. In this context, we herein present Q-raKtion, a systematic, semiautomated, flexible, and above all customizable approach that effectively aggregates information on biological activities retrieved by two of the most complete and widely used repositories, which are PubChem and ChEMBL.

The main advantages of the developed tool consist of (i) the availability of a general pipeline that integrates different data curation and integration tasks, (ii) the use of a graphical interface that allows the visual representation of each curation step, and (iii) the high propensity of the protocol to be easily interpretable and reproducible. Overall, Q-raKtion facilitates the access to bioactivity data curation for experts with different backgrounds, enabling the building of proprietary high-quality data sets for the specific needs of a research project. The pipeline is developed within KNIME,^18^ a user-friendly open-source platform that ensures a flexible and customizable module organization with other successful applications.^19,20^ Of note, the Q-raKtion tool can potentially be applied to build a high-quality data set of molecules that modulate any target of user interest.

We also report in this work a case study to practically illustrate how Q-raKtion can support data curation and integration. Particularly, given our interest in the kinase field,^21−23^ the developed pipeline was used to build a well-annotated, high-quality data set of AKT1 serine/threonine kinase inhibitors.

As a last note, we would like to emphasize the Q-raKtion workflow is freely available upon request for academic and noncommercial use.

## Methods

The Q-raKtion workflow was generated by using the open-source data analysis software KNIME^18^ (version 4.6.2 available free of charges at https://www.knime.com/), exploiting nodes from the KNIME Analytics Platform, KNIME Extensions, and Community Extensions by Cheminformatics toolkit as RDKit^24^ and Vernalis.^25^

## Results

This section provides general information on developing the workflow and the principles behind each step, while in-depth details are provided in the Supporting Information.

**Figure 1:**
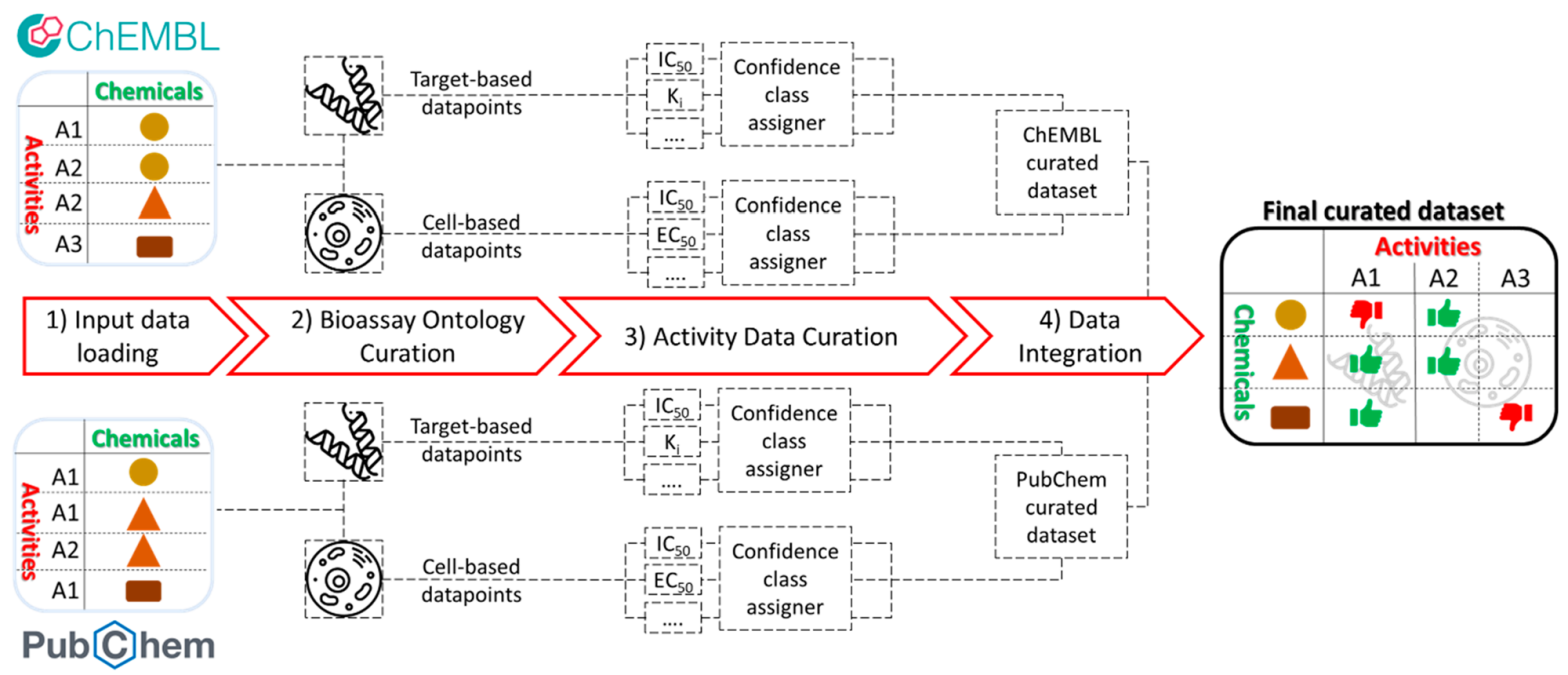
General scheme of the Q-raKtion workflow.

The Q-raKtion workflow is organized into four main steps (Figures 1 and S1): (1) input data loading, (2) bioassay ontology curation, (3) activity data curation, and (4) data integration.

### Step 1: Input Data Loading

The bioactivity data for molecules tested against a specific macromolecule need to be downloaded from the two previously mentioned databases as two separated datasheets, which are then imported into the Q-raKtion workflow. However, it is important to underline that these biological activities can belong either to different molecules or to the same compound, for which different types of biological measurements (e.g., IC_50_, *K*_i_, *K*_d_, etc.) or multiple data for the same type of activity (e.g., IC_50_-1, IC_50_-2, IC_50_-3) have been entered into the databases. In the latter case, the compound will be present in multiple rows, and each row will refer to one of the reported activities.

Each input datasheet is uploaded, read, and cleaned by filtering out duplicate rows. The processed data from ChEMBL and PubChem are then submitted in parallel to the bioassay ontology curation step (see also the Supporting Information).

### Step 2: Bioassay Ontology Curation

**Figure 2:**
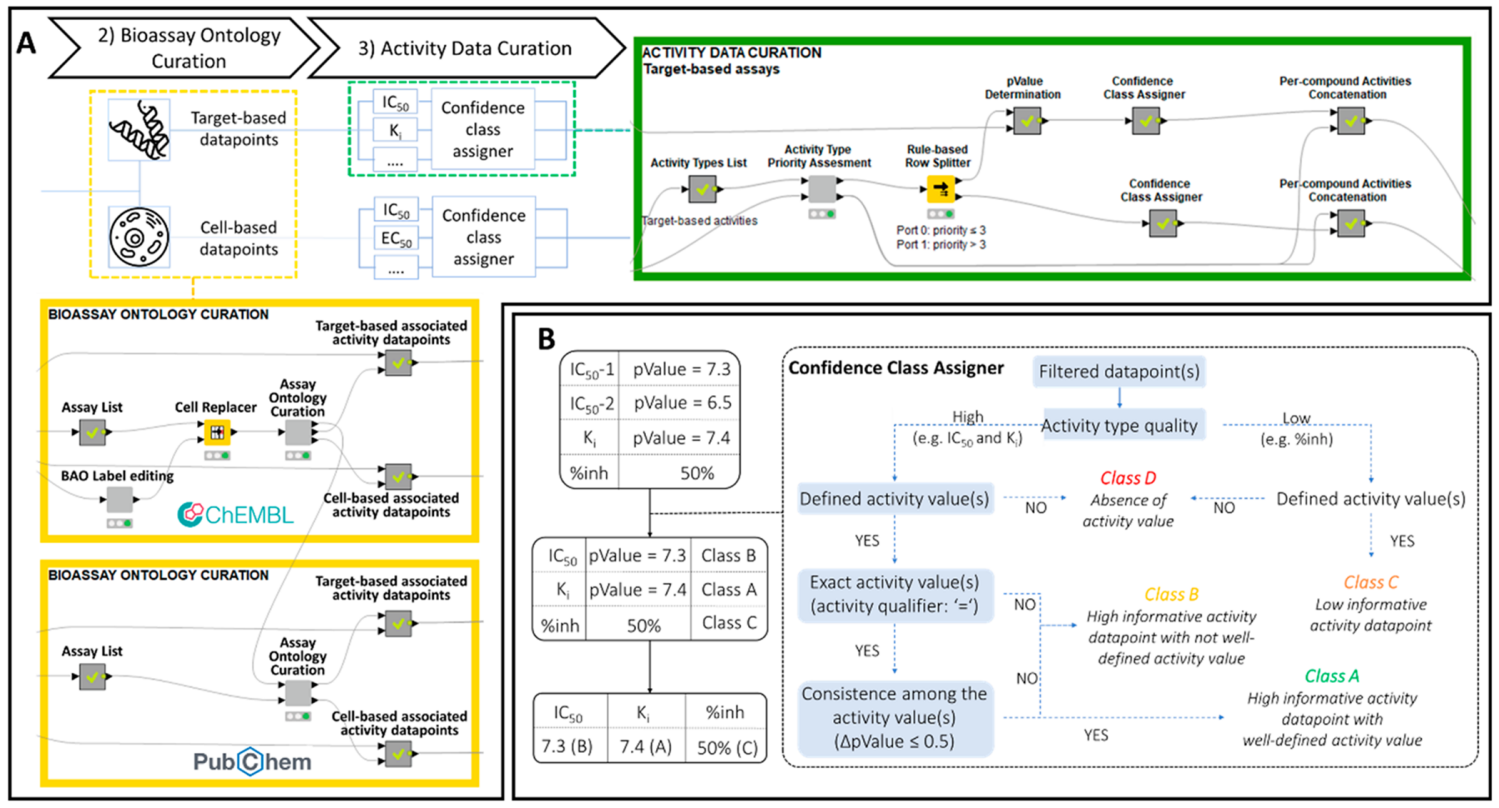
(A) Detailed overview of the bioassay ontology curation (yellow box) and activity data curation (green box) steps. (B) Schematic workflow of the procedure applied for the activity data curation step. The data points are filtered to retain all of the activities corresponding to the same compound (starting table). The data points with the same activity type are processed by the developed “Confidence Class Assigner” metanode that returns a row with the best activity datum coupled with the corresponding confidence class. All of the processed information is finally converted in a unique row. pValue is calculated as the negative log10 of the molar activity value; ΔpValue is calculated as the difference between the maximum and the minimum values of pValue. Examples of activity types include IC_50_, *K*_i_, and %inh (percentage of inhibition).

The final aim of this step is to distinguish, and accordingly to split, the data points collected in the previous step into two separated bioassay ontology classes, according to whether they were generated in target-based assays (where the compound is tested on the isolated target, such as biochemical and biophysical assays) or cell-based assays (where the whole cell is treated with the investigated compound). Even though both types of assays deliver crucial information on the activity of a specific compound on a certain target, given the different meanings of the data generated, they cannot be used together to develop predictive models. For this reason, the accurate data splitting according to the previously defined bioassay ontology criterium is obtained within the workflow in a stepwise manner (Figure 2A, yellow box). Using the combination of two properties for ChEMBL (i.e., “BAO label” and “Assay Description”) and one single property for PubChem (i.e., “aidname”), the workflow provides editable tables (Figure S2) as output of the “Assay Ontology Curation” components. At this point, each assay should be assigned by the user to the proper bioassay ontology class (see also the Supporting Information).

For each original datasheet, the activity data points are finally split according to the assigned ontology class (target-based associated activity data points and cell-based associated activity data points metanodes; Figure 2), thus generating a total of four different datasheets (two for both PubChem and CheEMBL) to be submitted to step 3.

### Step 3: Activity Data Curation

All of the multiple biological activities for the same compound are processed here through the following operations: (i) comparison between the different activity data points, (ii) selection of the best value for each activity type, (iii) assignment of the confidence class to the selected data point, and (iv) creation of a unique row that resumes all of the processed activities. All of these operations are illustrated in Figure 2 (see also the Supporting Information).

Going into the details, we derived a list of nonredundant activity types (e.g., IC_50_, *K*_i_, *K*_d_, etc.) by the metanode “Activity type list”. The “Activity type priority assessment” component creates an editable table that the user can fill to prioritize in a custom manner the importance of the activity types (Figure S3). As a general rule, high priority (priority ≤ 3) is assigned to the XC_50_ (e.g., IC_50_ and EC_50_) and the *K*_X_ (e.g., *K*_i_ and *K*_d_) measurements, while a lower priority (priority > 3) is used for less precise (e.g., % inhibition and % enzyme control activity) or misleading (e.g., activity, inhibition, and NULL) activity types.

The XC_50_ and *K*_X_ values are then converted into the corresponding pXC_50_ and p*K*_X_ values (−log10 of the original measurement; “pValue determination” metanode), and a list of nonredundant compounds (“GroupBy” node) is generated. Specifically, at this point in the workflow, we have developed a quality control protocol (the metanode “Confidence Class Assigner”) to label the single biological activity associated with a compound. Indeed, it is well-known that the performance of a predictive model strongly depends on the quality of the training data.^26^ The application of this quality control protocol involves the assignment of a confidence class that certifies the high quality of the activity measurement and its consistence with respect to other available data. Therefore, the activity type and the activity qualifier are (i.e., “=”, “>”, “≥”, “<”, or “≤”) used to assign to each data a confidence class ranging from A to D (Figure 2B). To assign the confidence class when more data are available for the same type of activity (IC_50_-1 and IC_50_-2) for a given compound, the same method is applied and integrated by calculating the difference between the corresponding maximum and minimum pValue (“ΔpValue” property).

The outputs of these operations are four datasheets (two for both PubChem and ChEMBL, reporting the target- and cell-based associated activity data points) where a row contains a unique molecule for which the best activity data point for each activity type is reported and flagged with the corresponding confidence class.

### Step 4: Data Integration

The target- and cell-based activity data points associated with the same compound identifier (as found in the “ChEMBL ID” and “PubChem CID” for ChEMBL and PubChem, respectively) are combined in a unique row, thus creating a comprehensive data set for ChEMBL and another separated one for PubChem.

These two data sets are finally merged in a unique final data set. However, given the absence of a common identifier, the workflow exploits the PubChem Identifier Exchange Service (available free of charge at https://pubchem.ncbi.nlm.nih.gov/idexchange/idexchange.cgi) to allow the user to retrieve the corresponding PubChem CID identifiers for the ChEMBL chemical structures.

In the final step, the comprehensive data sets of ChEMBL and PubChem are merged, and, for compounds having the same CID, the data points are collected in a unique row.

In the merging process, only the XC_50_ (i.e., IC_50_, EC_50_, and GI_50_) and *K*_X_ (i.e., *K*_i_ and *K*_d_) measurements are considered, as these are well-defined types of activity shared by both databases.

When two different activity values of the same type (e.g., IC_50_) are available from both ChEMBL and PubChem, only the best data point is retained, and a second round of confidence class assignment is performed to update the confidence class. Additionally, during this round, an extra rule is added to assign the “A” confidence class to all compounds with “inactive” flag in PubChem and no activities reported on ChEMBL or PubChem (see also the Supporting Information).

## Example of Application on the AKT1 Protein

As a case study, we focused our attention on the serine/threonine protein kinase AKT1 (also known as PKB). AKT1 is a well-validated therapeutic target for cancer,^27^ being a key component of the pI3K/AKT/mTOR signaling pathway.

Currently, only a few compounds able to exert an inhibitory activity against AKT1 have entered clinical evaluation,^28^ with no inhibitors approved by the Food and Drug Administration (FDA) for clinical use. Conversely, many small molecules able to modulate AKT1 have been reported over the years.

At first, the bioactivity data regarding compounds tested against this kinase were downloaded from ChEMBL and PubChem (see also the Supporting Information) and submitted to the Q-raKtion workflow. For both databases, the number of data points exceeded the count of unique compounds only to a small extent. In detail, from PubChem we collected 365 354 data points for 362 293 unique compounds, whereas in ChEMBL about 1.3 data points were found for each compound (8792 data points relating to 6480 unique compounds). It is well-known that PubChem is routinely updated importing data points from a wide range of data sources, including ChEMBL. Indeed, in our specific case, 8426 PubChem data points (corresponding to 5833 unique compounds) came from ChEMBL, as indicated in the PubChem property “aidsrcname”.

The emerged data points per compound ratio pointed out that most compounds had only a single associated activity (Table 1).

**Table 1:** Summary of the Per Compound Data Points Distribution for the ChEMBL and PubChem Compounds

no. of data point(s)	ChEMBL molecules	PubChem molecules	no. of data point(s)	ChEMBL molecules	PubChem molecules
1	4942	360356	13	2	1
2	1260	1618	16	3	2
3	129	157	18	1	1
4	71	81	20	1	1
5	22	17	22	0	1
6	13	15	32	1	0
7	7	10	35	0	1
8	17	17	45	1	1
9	5	6	109	0	1
10	4	4	174	0	1
12	1	2	total	6480	362293

Interestingly, the remarkable difference in the quantity of molecules between the ChEMBL and PubChem data sets (6480 vs 362 293, respectively) was determined by the number of compounds with only one associated data point (360 356 and 4942 compounds for PubChem and ChEMBL, respectively), while a comparable number of unique compounds having at least two data points was found (1936 and 1538 for PubChem and ChEMBL, respectively).

Considering the type of biological activity reported by the data points, ChEMBL and PubChem shared eight flags as illustrated in Table 2A. In all cases but GI_50_, the two repositories provided a different amount of information for the same activity type with ChEMBL showing more data points than PubChem. This observation seemed to be in contrast with the different original data point size of the two databases previously underlined, but it was explained by observing that 360 255 PubChem data points presented the “NULL” label in the activity type property (Table 2B). In the analyzed data, the “NULL” label was associated with primary screenings with 99.2% of the corresponding data points referred to a unique assay, that is, to the AKT1 primary screening performed by the National Center for Advancing Translational Science (AID: 651550).

**Table 2:** Summary of the Number of Data Points and Unique Compounds for the Different Types of Biological Activities Common to (A) or Distinctive between (B) ChEMBL and PubChem

(A)	ChEMBL		PubChem	
activity type	no. of data points	no. of unique compounds	no. of data points	no. of unique compounds
IC_50_	4039	3327	4068	3331
*K*_d_	552	394	841	400
*K*_i_	851	824	106	103
EC_50_	5	3	12	5
GI_50_	14	7	14	7
activity	591	431	45	29
inhibition	2200	1617	12	12
ratio	45	12	1	1
total	8297	6615	5099	3888

(B)	ChEMBL		PubChem	
activity type	no. of data points	no. of unique compounds	no. of data points	no. of unique compounds
NULL	NA^a^	NA^a^	360255	358770
% control	110	110	NA^a^	NA^a^
Delta Tm	41	37	NA^a^	NA^a^
FC	13	8	NA^a^	NA^a^
residual activity	331	167	NA^a^	NA^a^
total	495	322	360255	358770

a NA, not available.

As was already mentioned, in the “Bioassay ontology curation” step, two ontology classes were defined (i.e., target- or cell-based assays) and accordingly used to classify the available bioassays.

For AKT1, ChEMBL classified the bioassays with four different types of BAO labels, named single protein format (674 assays corresponding to 5715 data points), cell-based format (416 assays corresponding to 1791 data points), subcellular format (1 assay corresponding to 242 data points), and assay format (96 assays corresponding to 1043 data points).

While it was clear that the single protein format and the cell-based format could be classified as target- and cell-based assays, respectively, how to classify both the subcellular format and the assay format was less obvious. For this reason, the descriptions of the latter were manually checked in an attempt to assign them the proper ontology class.

Where the description of the assay was still too vague (i.e., some assays were described with the very general sentence “inhibition of AKT1”), precise information was retrieved from the original paper. This exhaustive analysis led to classifying the previously ambiguous ChEMBL data as 795 target-based and 392 cell-based assays.

Additionally, the ChEMBL assays labeled as single protein-format and cell-based format were double checked as well. We found that in a few cases the BAO label did not perfectly correspond to our classification in target-based and cell-based assays (Table S1). This observation highlighted the need for the user to verify that the available flags are in line with the specific objectives of the research project.

Regarding PubChem, 1276 unique assays were associated with data points related to molecules tested against AKT1, but these were not labeled in a way that would support the ontology classification. Therefore, it was necessary to follow the same process described above for ChEMBL data to obtain a correct ontology classification, that is, 820 and 456 target- and cell-based assays, respectively.

It is worth emphasizing that, in general, this step provides a quick view on the most frequently used assays to investigate the activity of molecules against the explored target; for example, the resulting information can be particularly useful in the case of cell-based assay to get clues about the most utilized cell lines (Table S2).

Finally, it was interesting to note that only a tiny fraction of the collected compounds in each database presented bioactivity data in both target- and cell-based assays (350 out of 6480 in ChEMBL; 378 out of 362 292 in PubChem). Once more, despite the difference in the total amount of downloaded compounds from ChEMBL and PubChem, the number of well-characterized molecules was comparable.

**Figure 3:**
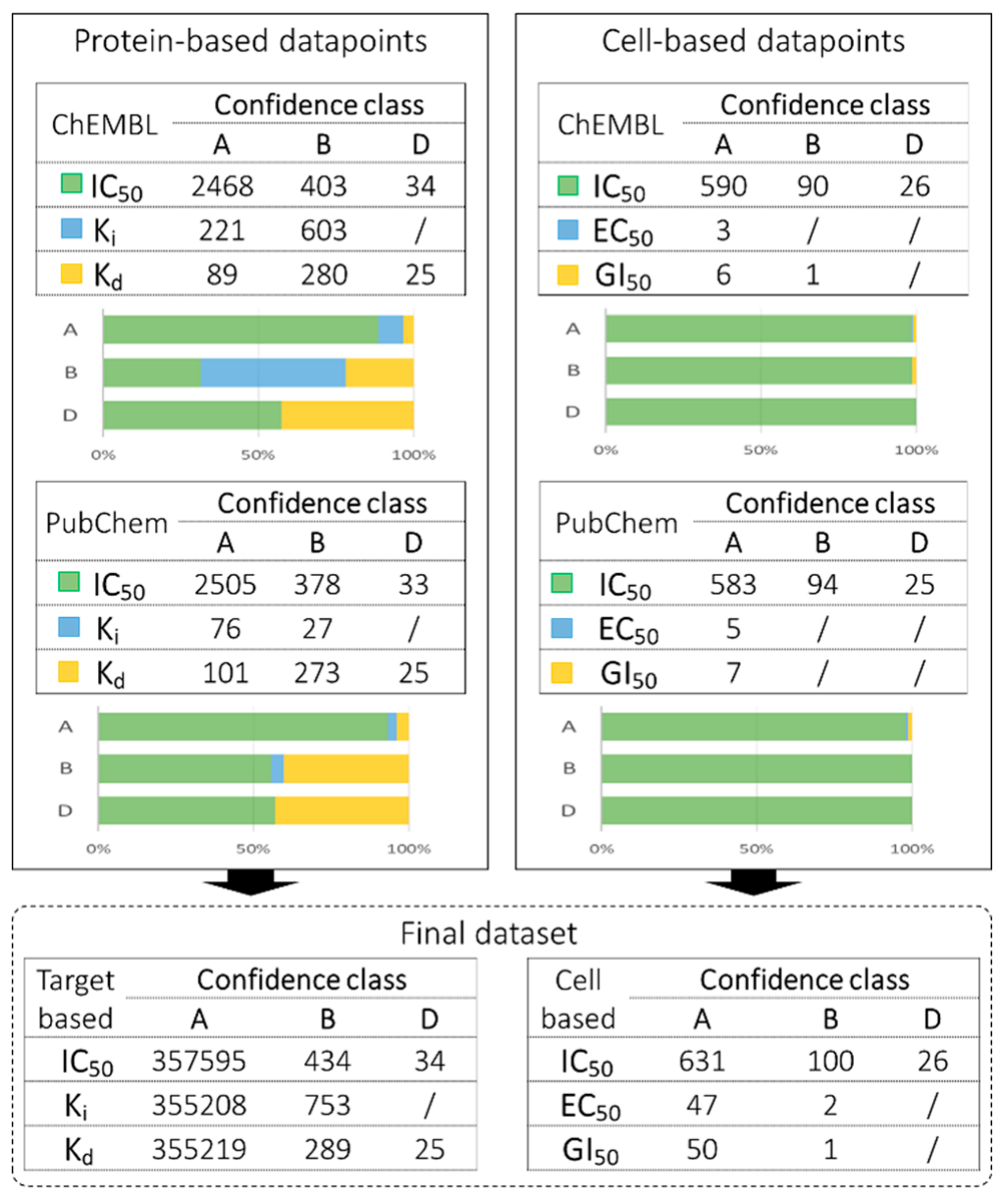
Number of data points collected in each confidence class for the high-quality activity types (i.e., XC_50_ and *K*_X_) both in the curated ChEMB and PubChem databases and in the final data set of AKT1 compounds.

The “Activity data curation” step produced a comparable number of activity data points between the two repositories for all the activity types except *K*_i_ (Figure 3). Yet for the target- and cell-based data points, most of the activity data came from the IC_50_ values, with the majority of them collected in the confidence class A. Noteworthy, in PubChem and ChEMBL, the IC_50_ values can refer to activities measured in target- or cell-based assays. In this regard, the ontology curation step appears critical to correctly split these types of data points.

Finally, the Q-raKtion workflow aided the generation of a data set containing 362 988 compounds tested on AKT1, 358 109 of which have the highest confidence class A for at least one activity measurement.

## Conclusions

We herein present the Q-raKtion tool, a KNIME workflow freely available to researchers to aid the curation and integration of activity data points provided by the public databases PubChem and ChEMBL. Specifically, Q-raKtion guides the user through the navigation of bioactivity data annotations to (i) fix potential inconsistencies (e.g., incorrect ontology annotations) and (ii) set up a confidence class system to prioritize activity information.

No specific knowledge of KNIME software is required to use the proposed workflow, and the user-selected settings are saved at each step, thus creating a pipeline that can be easily repeated later in a fully automated manner. Therefore, although the first time the pipeline is used it is necessary to set some parameters through a manual work, once the workflow has been set up for a target, the user has a fully automated tool that can be reused (e.g., data set update operations with new data) with significant savings in time/resources as compared to the same work done with a fully manual approach.

The flexibility of Q-raKtion and its integration with other third-party software and tools allow this workflow to be used as a starting point for customizations that meet a wide range of specific needs, such as integration of PubChem and ChEMBL information with additional proprietary or public data points, or the use of the generated high-quality data set to train predictive models using classical strategies (e.g., QSAR studies) or more advanced artificial intelligence algorithms (e.g, machine learning).

## Data and Software Availability

The Q-raKtion workflow and all of the files generated by the curation of AKT1 activity data points are freely available upon request for academic and noncommercial use.

## Supporting Information Available

(1) Computational methods: additional details on steps 1–4 of the Q-raKtion workflow; (2) details on the procedure applied to download the AKT1 data points; and (3) supplementary figures and tables (AKT1-files_Dec2021): files produced within the application of the Q-raKtion workflow for the AKT1 protein (PDF)
