# Supplementary material for: Q-raKtion: A Semiautomated KNIME Workflow for Bioactivity Data Points Curation
Source: J Chem Inf Model. 2022 Nov 28;62(24):6309–15. doi: 10.1021/acs.jcim.2c01199 (PMC9795488; doi:10.1021/acs.jcim.2c01199)
Supplement: Supplementary file 1 — ci2c01199_si_001.pdf [file ci2c01199_si_001.pdf]

Supporting Information

Q-raKtion: A semi-automated KNIME workflow for bioactivity datapoint curation

Deborah Palazzotti, [1] Martina Fiorelli, [1] Stefano Sabatini, [1] Serena Massari, [1] Maria Letizia Barreca, *,[1] and Andrea Astolfi *,[1]

[1] Department of Pharmaceutical Sciences, "Department of Excellence 2018-2022", University of Perugia, Via del Liceo, 1, 06123, Perugia, Italy

To whom corresponding should be addressed: maria.barreca@unipg.it, andrea.astolfi@unipg.it

Table of Contents

1. Computational Methods

1.1 **Step 1: Input data loading**

1.2 **Step 2: Bioassay ontology curation**

1.3 **Step 3: Activity data curation**

1.4 **Step 4: Data Integration**

2. Collection of AKT1 activity datapoints from ChEMBL and PubChem Data

3. Supporting Figures and Tables

3.1 **Figure S1**

3.2 **Figure S2**

3.3 **Figure S3**

3.4 **Table S1**

3.5 **Table S2**

1. Computational Methods

1.1 Step 1: Input data loading

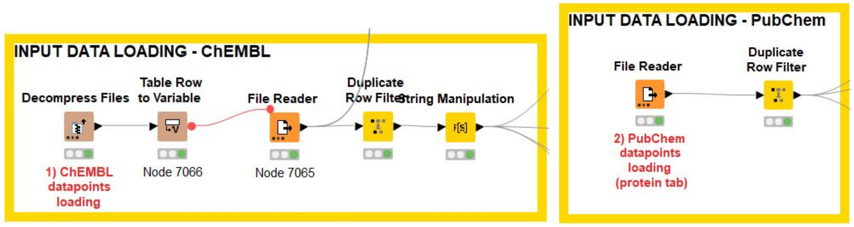

The raw data, downloaded from ChEMBL and PubChem repositories, are uploaded into the workflow in the *Decompress Files* and *File Reader* nodes, respectively. Both files presented duplicated rows that are deleted by using the *Duplicate row filter*. Only for the ChEMBL datapoints, the column "Standard relation" is manipulated to remove the quote character (*String Manipulation* node).

1.2 Step 2: Bioassay ontology curation

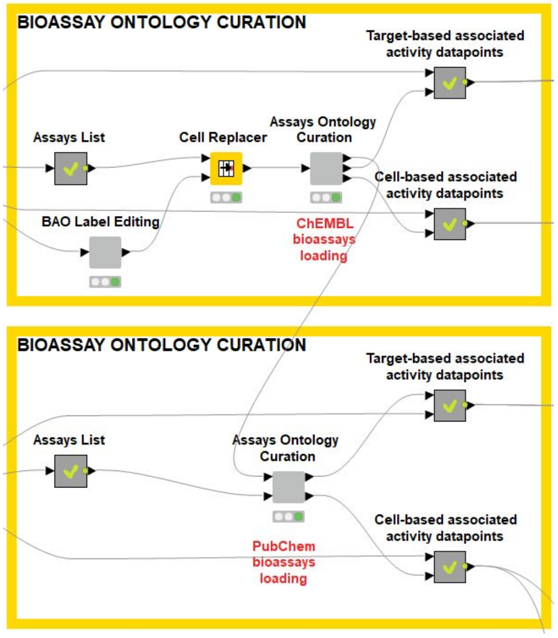

In the pre-filtered datasheets, each biological datapoint is coupled with an assay identifier corresponding to the specific assay performed to obtain that data, named 'Assay ChEMBL ID' and 'Aid' for ChEMBL and PubChem, respectively. Using these properties, two of non-redundant assays lists (one for each original repository) is obtained by applying the metanode *Assays list*. The generated assays lists are submitted to bioassay ontology classification.

The bioassay ontology classification can be performed in three different ways. Importantly, based on the selected classification procedure, the connectivity of the "Assay Ontology Curation" component needs to be modified.

By using the BAO label. In ChEMBL each datapoint is associated with the corresponding ChEMBL BioAssay Ontology label (or "BAO label") flag. The "BAO label" describes and organize the biological screening assays for data analysis. Typical "BAO label" can be "single protein format" or "cell-based format" flags. However, the "BAO label" can currently correspond to over 700 different flags. In the *BAO label editing* component the user can assign the correct bioassay ontology class to each "BAO label" flag reported in the ChEMBL datasheet

(Figure S2). Then, the workflow assigns the correct ontology class according to the assay "BAO label" (*Cell Replacer* node). For example, assigning the "target-based assay" class to the "single protein format" "BAO label" flag, all the assays having as "BAO label" the "single protein format" flag are accordingly assigned to the "target-based assay" class (see Figure below).

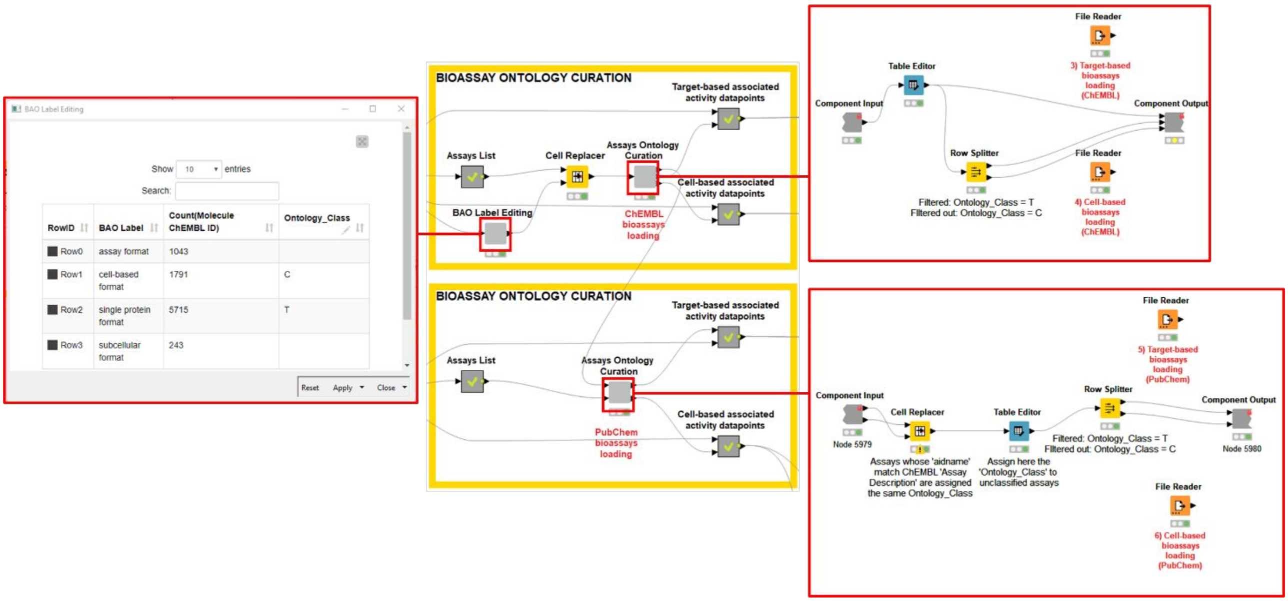

For the list of assays obtained from PubChem, the bioassay ontology class needs to be assigned manually according to the "aid name" property that provide a brief description of the bioassay. Of note, the same property (named "Assay Description") can be found in ChEMBL. Thus, assays having an identical assay description in ChEMBL and PubChem can be assigned to the same ontology class based on the ChEMBL "BAO label" flag.

By using manual assignment. Given the key role that correct data plays in the final performance of a predictive model, a manual check of the proper classification is highly recommended. This step can be performed within the A*ssay ontology curation* component (right click with the mouse to select "Interactive view"; see figure below for the interactive interface).

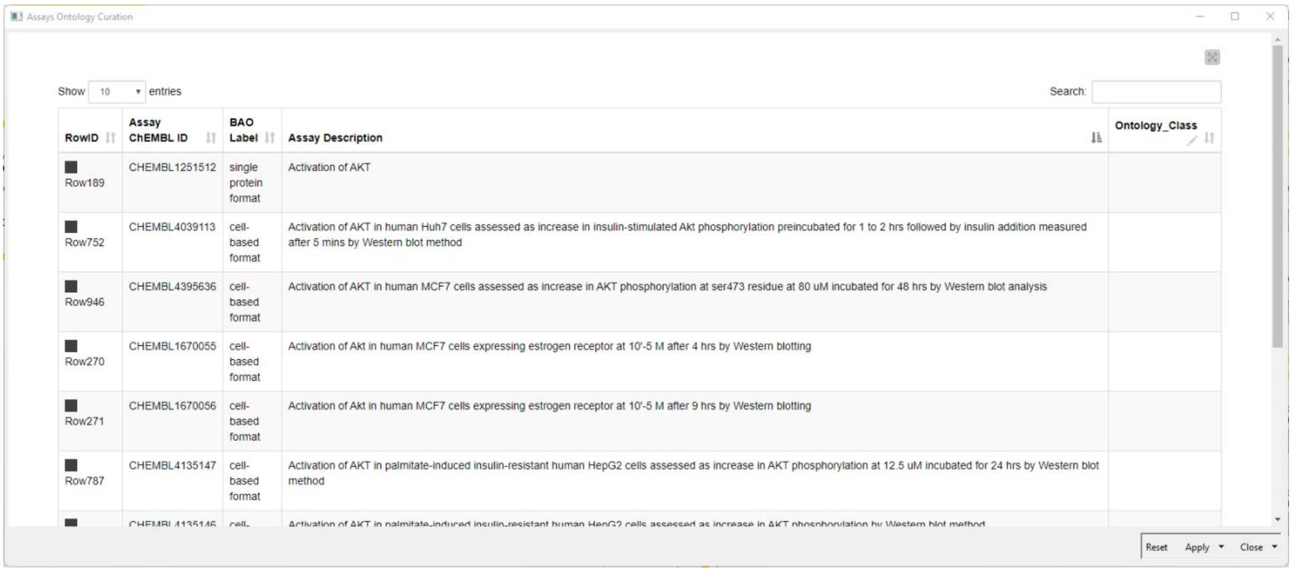

Now the user can check the bioassay description and correct/assign the proper class. To confidently assess the correct assay ontology when the description is not clear, the original publication(s) can be checked.

By using external files. Alternatively, the list of assays can be split externally in csv files containing the list of target-based and cell-based assays. These two files can be then loaded to the workflow (see figure below).

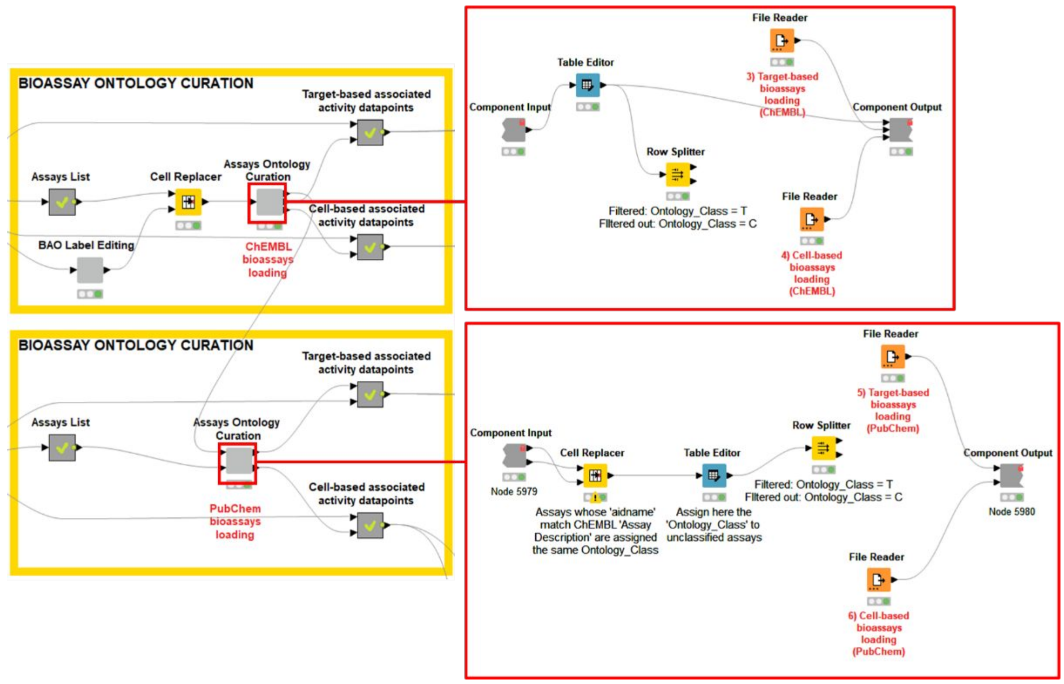

Finally, the datapoints are split accordingly to the assigned ontology class (*Target-* and *Cell-based associated activity datapoints* metanodes).

1.3 Step 3: Activity data curation

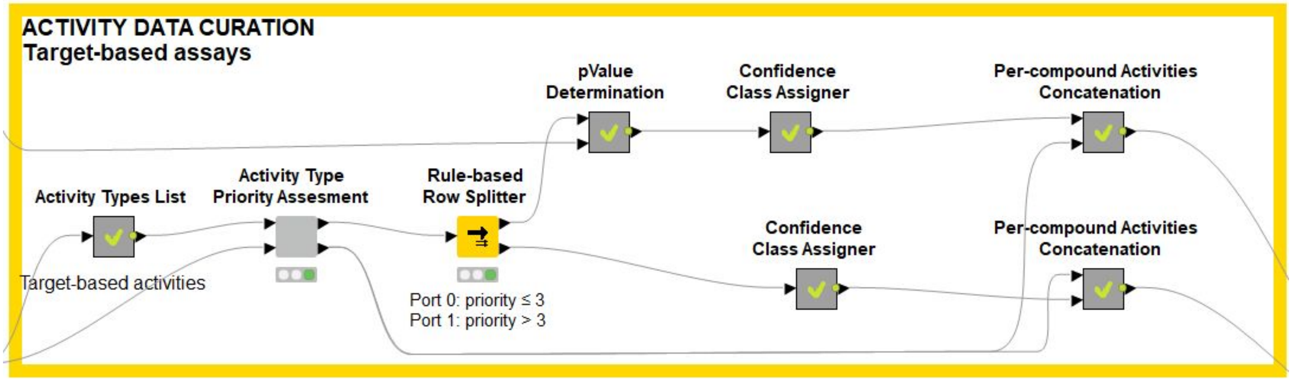

Firstly, a list of non-redundant activity types (e.g. IC_{50}, K_i, K_d, etc.) is derived by using the 'Standard Type' and 'acname' properties for ChEMBL and Pubchem (metanode *Activity type list*), respectively.

Secondly, we have developed a protocol to insert a quality label on biological activity associated to a compound. Indeed, it is well known that the performance of a predictive model strongly depends on the quality of the training data. The application of this quality control protocol results in the assignment of a confidence class that indicates the quality of the activity measure and its consistence with respect to other available data.

A numerical priority value (*Activity-type priority assessment* component) can be assigned by the user to discriminate high from low informative activity types. As default setting, high priority (priority $\leq$ 3) are assigned to XC_{50} (i.e. IC_{50}, EC_{50} and GI_{50}) and K_X (i.e. K_i and K_d) measurements, while a lower priority (priority > 3) is used to less precise (e.g. % inhibition, % enzyme control activity) or to misleading (e.g. Activity,

Inhibition, NULL) activity types. The datapoints are splitted based on the assigned activity priority (*Rule-based row splitter* node), and thus high and low informative activity types are processed separately.

The XC_{50} and K_X values are then converted into the corresponding pXC_{50} and pK_X values ($-log_{10}$ of the original measure; *pValue determination* metanode) to allow datapoints comparison regardless the order of magnitude (i.e. μM or nM). This step coupled to the previous classification (and datapoints splitting) in target-based and cell-based assays, ensures that all the data provided as input in the next steps (e.g. to the metanode *Confidence Class Assigner*) could be compared in terms of assay ontology and activity pValue.

At this point, a list of non-redundant compounds is generated and for each of them all the available datapoints are analyzed. We have developed the metanode *Confidence Class Assigner* to automatically determine the quality of the analyzed datapoint. Specifically, based on the activity types (e.g. IC_{50}, K_i, K_d, etc, collected in the “Standard relation” and “acvalue” columns for ChEMBL and PubChem, respectively) and activity qualifier (i.e. “=”, “>”, “≥”, “<” or “≤” collected in the “Standard relation” and “acvalue” columns for ChEMBL and PubChem, respectively), the metanode automatically assign a confidence class that can range from A to D.

When more than one data is available for the same activity type (IC_{50}-1, IC_{50}-2) for a given compound, only the best activity value is retained and the data consistence is assessed by calculating the difference between the maximum and minimum corresponding pValue ("ΔpValue" property); The activity type, the activity qualifier (i.e. “=”, “>”, “≥”, “<” or “≤”) and the "ΔpValue" property are then used to assign a datapoint confidence class. Each row that the *Confidence Class Assigner* metanode provides as output refers to a single activity value for a single activity type of a given compound coupled with the proper confidence class.

The multiple annotated activities corresponding to the same compound are then aggregated in a unique row (*Per-compound Activities Concatenation* metanode/*Concatenate* node/*GroupBy* node). The outputs of these operations are four datasheets corresponding to two datasheets for both PubChem and CheEMBL, respectively, reporting the target-based and cell-based associated activity datapoints. In these tables, a row contains a unique molecule for which the best activity datapoint of each activity type is reported and flagged with the corresponding confidence class.

1.4 Step 4: Data integration

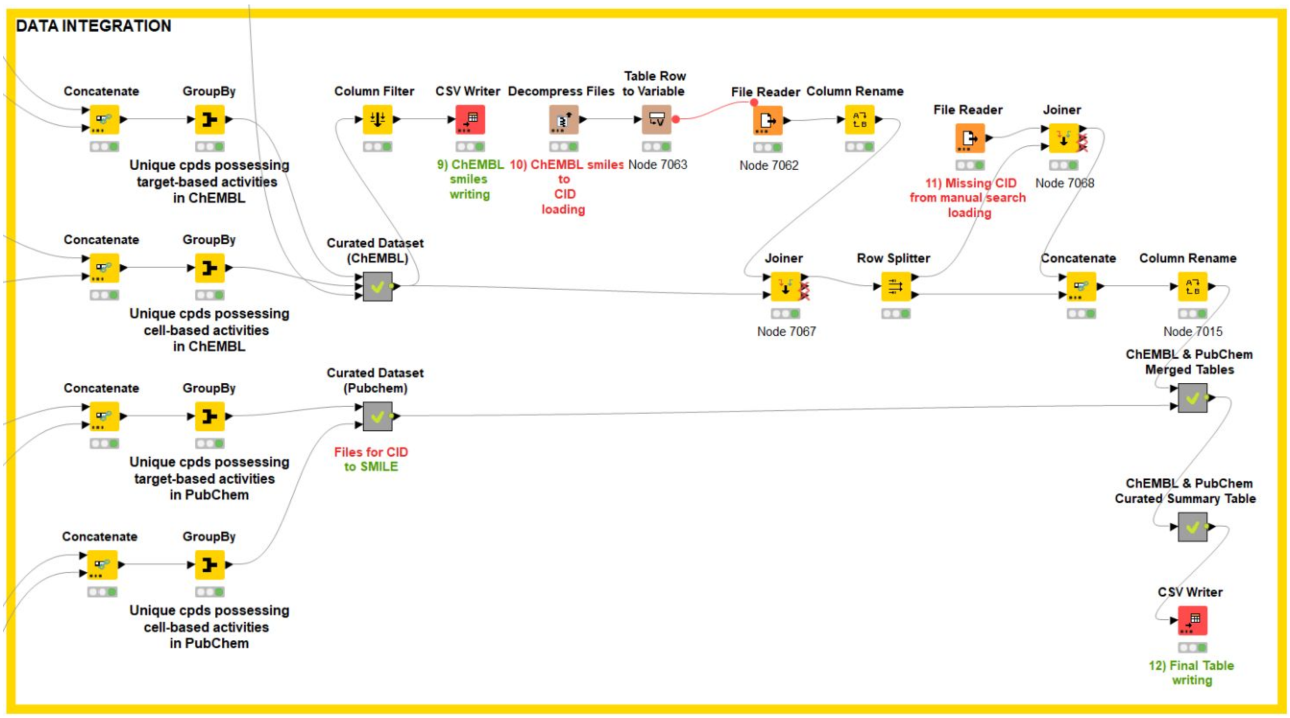

At first, two separated and comprehensive datasets of ChEMBL and PubChem are created (*Curated Dataset (ChEMBL)* and *Curated Dataset (PubChem)* metanodes). Towards this aim, the target-based and cell-based activity datapoints associated to the same compound identifier (as found in the "ChEMBL ID" and "PubChem CID" for ChEMBL and PubChem, respectively) are combined in a unique row.

These two datasets are finally merged in a unique final dataset. However, given the absence of a common identifier, the workflow exploits the PubChem Identifier Exchange Service (available free of charge at https://pubchem.ncbi.nlm.nih.gov/idexchange/idexchange.cgi) to allow the user to retrieve the corresponding PubChem CID identifiers for ChEMBL chemical structures. Specifically, the workflow generates a file with the smile structures of ChEMBL compounds (*CSV writer* node). This file can be uploaded in the previously mentioned webpage selecting "SMILES" as Input ID list option. This search returns a table containing the input smiles and the corresponding CIDs that can be uploaded into the workflow using the *Decompress Files* node, thus allowing the assignment of the proper PubChem identifier (i.e. CID) to each ChEMBL compound. The PubChem Identifier Exchange Service can return a result in which some compounds are missing. The CIDs of the missing compounds can manually search and provided to the workflow using the second *File reader – missing CIDs* node. In the case of workflow application on AKT1 protein, the ChEMBL IDs of the missing compounds were used as query in the PubChem Identifier Exchange Service instead of the smiles structure. The service provided a list of synonyms for each ChEMBL ID, among which the PubChem CID was reported. Starting from this output a file reporting the ChEMBL ID and the corresponding CID was created and uploaded.

In the final step, the comprehensive datasets of ChEMBL and PubChem are merged, and, for compounds having the same CID, the datapoints are collected in a unique row (*ChEMBL & PubChem Merged Tables* metanode).

In the merging process, only the XC_{50} (e.g. IC_{50}, EC_{50} and GI_{50}) and K_X (e.g. K_i and K_d) measurements are considered, as these are high informative activity types shared by both databases (*ChEMBL & PubChem Curated Summary Table* metanode). When two different activity values of the same type (e.g. IC_{50}) are available both from ChEMBL and PubChem, only the best datapoint is retained and the confidence class is updated applying the following rules in a stepwise manner:

- When both activities are in A confidence class and the "ΔpValue" is ≤ 0.5, the highest value is retained, and the A flag is assigned.
- When both activities are in A confidence class and the "ΔpValue" is ≥ 0.5, the highest value is retained,and the B flag is assigned.
- When one of the two activities is in D confidence class, the highest value is retained, and the D flag is assigned.
- When one of the two activities is in B confidence class, the highest value is retained, and the B flag is assigned.
- When a compound is flagged as "inactive" in the "activity" property in the PubChem original datasheet and no additional activities are reported on ChEMBL or PubChem, the compound is flagged as truly inactive and assigned to the A confidence class.

2. Collection of AKT1 activity datapoints from ChEMBL and PubChem Data

The UniProt ID P31749, corresponding to the AKT1 protein, was used to query the web interfaces of ChEMBL and PubChem databases (access date December 2021).

From ChEMBL 8795 datapoints were downloaded selecting the target ID CHEMBL4282 single protein (https://www.ebi.ac.uk/chembl/g/#browse/activities/filter/target_chembl_id%3ACHEMBL4282). From this webpage all the available activities were selected and downloaded as csv file.

Within PubChem, the search produced two results, one associated to the AKT1 gene page and one to the AKT1 protein page. Only the results concerning the protein tab were used for this work. From the PubChem webpage of the AKT1 protein (RAK-alpha serine/threonine-protein kinase https://pubchem.ncbi.nlm.nih.gov/protein/P31749#section=Chemicals-and-Bioactivities), we selected and downloaded all the tested compounds in the Chemicals and Bioactivities section as csv file. In total, 366895 bioactivity datapoints were downloaded.

3 Supporting Figures and Tables

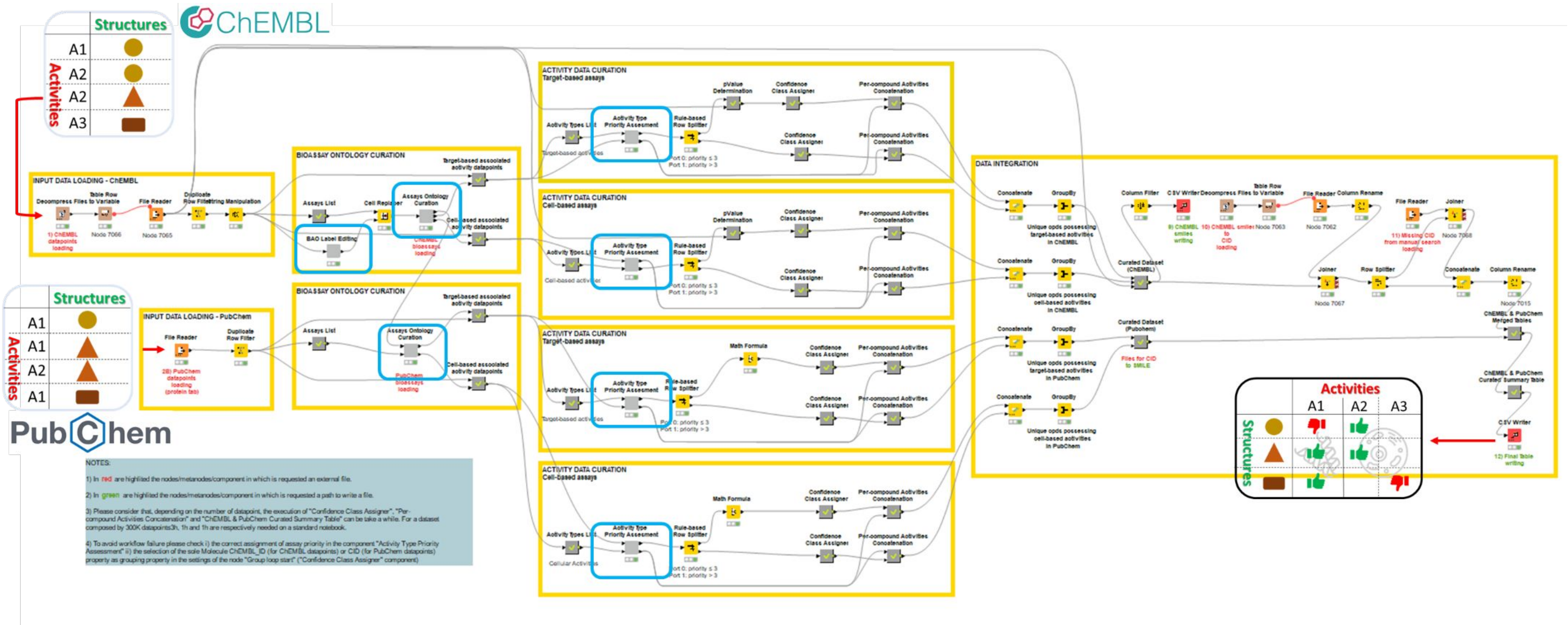

Figure S1. Detailed view of the Q-raKtion organization. The input tables downloaded from ChEMBL and PubChem are loaded into the workflow (“Input data loading”: red rows, left part), and through automated and manually-assisted (blue box) data curation steps, Q-raKtion returns the final dataset of compounds with the corresponding annotated activities (red row, right part).

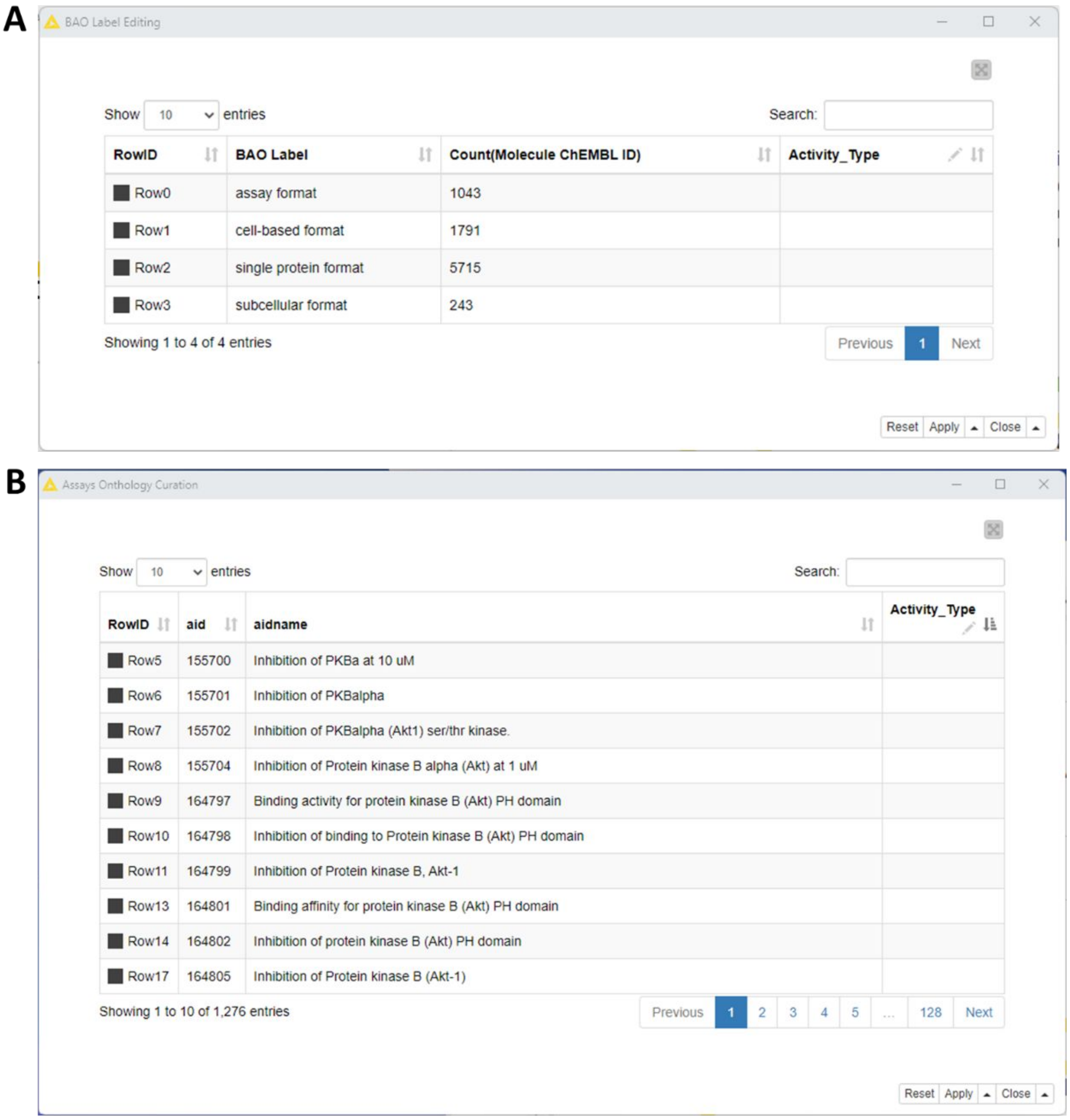

Figure S2. Example of application on AKT1 protein. The "BAO label editing" and "Assay Ontology Curation" components provide the interactive tables for the manual curation of the bioassays ontology illustrated in panel A and B, respectively. The column Ontology Class is editable to manually assign the proper bioassays ontology class based on the "BAO label" column or the assay description property ("aidname" column for PubChem, "Assay Description" column for ChEMBL).

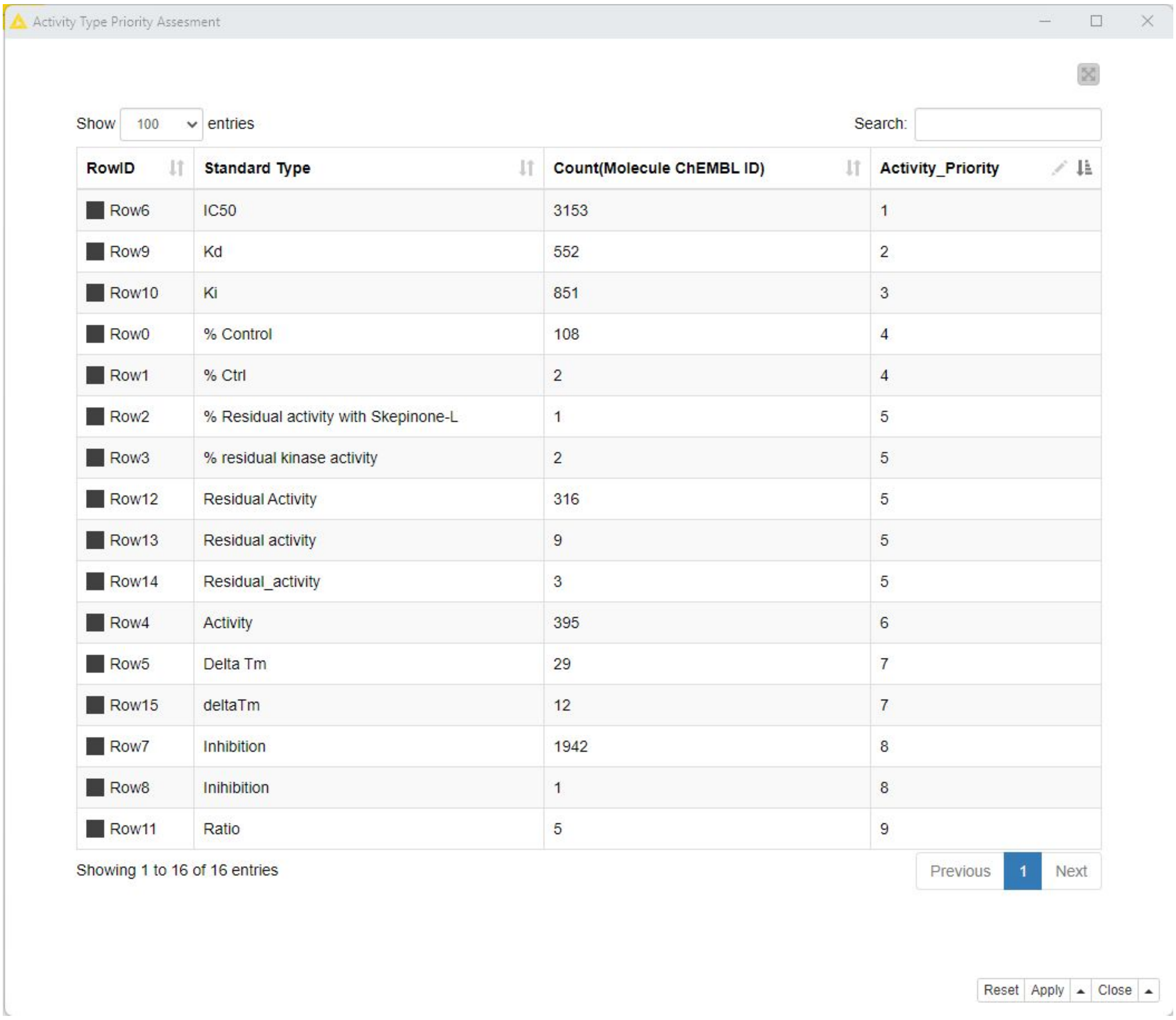

RowID	Standard Type	Count(Molecule ChEMBL ID)	Activity_Priority
Row6	IC50	3153	1
Row9	Kd	552	2
Row10	Ki	851	3
Row0	% Control	108	4
Row1	% Ctrl	2	4
Row2	% Residual activity with Skepinone-L	1	5
Row3	% residual kinase activity	2	5
Row12	Residual Activity	316	5
Row13	Residual activity	9	5
Row14	Residual_activity	3	5
Row4	Activity	395	6
Row5	Delta Tm	29	7
Row15	deltaTm	12	7
Row7	Inhibition	1942	8
Row8	Inihibition	1	8
Row11	Ratio	5	9

Figure S3. Example of application on AKT1 protein. The "Activity-type priority assessment" component provides the illustrated interactive table for the ChEMBL datapoints corresponding to AKT1 activities. The Activity Priority column is editable and can be used i) to discriminate high informative from low informative activity types and ii) to aggregate identical activity types (*e.g.* "% Control" and "% Ctrl")

Table S1. Example of application on AKT1 protein. List of the assays to which we assigned a different bioassay ontology class with respect to the original BAO label

Assay_ChEMBL_ID	Assay_Description	Bioassay ontology class	Number of datapoints	BAO label
CHEMBL1244112	Inhibition of bFGF-induced Akt phosphorylation in HUVEC at 100 nM treated 2 hrs before bFGF challenge by Western blotting	Cell-based assay	1	single protein format
CHEMBL1244115	Inhibition of VEGF-induced Akt phosphorylation in HUASMC at 100 nM treated 2 hrs before VEGF challenge by Western blotting	Cell-based assay	1	single protein format
CHEMBL1244116	Inhibition of bFGF-induced Akt phosphorylation in HUASMC at 100 nM treated 2 hrs before bFGF challenge by Western blotting	Cell-based assay	1	single protein format
CHEMBL2167409	Inhibition of TNFalpha-stimulated Akt1 phosphorylation at Ser473 assessed as pAKT level in HUVEC at 10 uM pretreated for 16 hrs before TNFalpha challenge measured after 6 hrs by Western blot analysis (Rvb = 249%)	Cell-based assay	1	single protein format
CHEMBL2167425	Inhibition of TNFalpha-stimulated Akt1 phosphorylation at Ser473 in HUVEC at 10 uM pretreated for 16 hrs before TNFalpha challenge measured after 15 mins by Western blot analysis	Cell-based assay	1	single protein format
CHEMBL3540286	Induction of AKT phosphorylation at Ser473 in neonatal HDF at 10 uM by Western blot analysis	Cell-based assay	1	single protein format
CHEMBL3880120	Induction of anti-CD3-stimulated Akt phosphorylation in human PBMC preincubated for 15 mins followed by anti-CD3 stimulation measured after 5 to 15 mins by phosflow method	Cell-based assay	1	single protein format
CHEMBL4045631	Inhibition of AKT1 labeling at ATP binding site (GTFGKVILVK) in human PBMC at 1 uM	Cell-based assay	1	single protein format
CHEMBL4045874	Inhibition of AKT1 labeling at ATP binding site (GTFGKVILVK) in human PBMC at 0.1 uM	Cell-based assay	1	single protein format
CHEMBL889652	Inhibition of IL8-induced Akt phosphorylation in human neutrophils at 0.06 uM	Cell-based assay	1	single protein format
CHEMBL889653	Inhibition of IL8-induced Akt phosphorylation in human neutrophils at 0.01 uM	Cell-based assay	1	single protein format
CHEMBL889654	Inhibition of IL8-induced Akt phosphorylation in human neutrophils at 0.05 uM	Cell-based assay	1	single protein format
CHEMBL947433	Inhibition of Akt1 by cellular assay	Cell-based assay	28	single protein format
CHEMBL1038874	Binding affinity to human AKT1 at 50 nM by cell-based competition binding assay relative to control in presence of DTT	Target-based assay	1	cell-based format
CHEMBL1045159	Binding affinity to human AKT1 at 500 nM by cell-based competition binding assay relative to control in presence of DTT	Target-based assay	1	cell-based format
CHEMBL1048631	Binding affinity to human AKT1 at 200 nM by cell-based competition binding assay relative to control in presence of DTT	Target-based assay	1	cell-based format

CHEMBL1068863	Inhibition of human recombinant AKT1 expressed in Sf21 cells at 10 uM	Target-based assay	1	cell-based format
CHEMBL1247620	Inhibition of human PKBalpha expressed in SF9 cells at 10 uM	Target-based assay	9	cell-based format
CHEMBL1249532	Inhibition of human PKBalpha expressed in SF9 cells at 20 uM	Target-based assay	3	cell-based format
CHEMBL1249556	Inhibition of human PKBalpha expressed in SF9 cells at 50 uM	Target-based assay	2	cell-based format
CHEMBL1249580	Inhibition of human PKBalpha expressed in SF9 cells at 1 uM	Target-based assay	11	cell-based format
CHEMBL1249802	Inhibition of human PKBalpha expressed in SF9 cells	Target-based assay	2	cell-based format
CHEMBL1819981	Inhibition of AKT1 expressed in Escherichia coli or baculovirus-infected insect cells using gamma-[33P]ATP at 400 uM by scintillation proximity assay	Target-based assay	17	cell-based format
CHEMBL1820096	Inhibition of AKT1 expressed in Escherichia coli or baculovirus-infected insect cells by scintillation proximity assay	Target-based assay	5	cell-based format
CHEMBL1961273	Inhibition of human recombinant AKT1 expressed in Sf9 cells using GSK3(14-27) as substrate after 80 mins by scintillation counting	Target-based assay	2	cell-based format
CHEMBL2066179	Inhibition of human recombinant Akt1/PKBalpha expressed in insect cells using CREBtide as substrate assessed as residual activity at 1 uM after 60 mins by Cerep assay	Target-based assay	1	cell-based format
CHEMBL2183039	Inhibition of Akt1 (1 to 480) expressed in Sf9 cells by indirect affinity mass spectrometry assay	Target-based assay	5	cell-based format
CHEMBL2183040	Inhibition of Akt1 (1 to 480) expressed in Sf9 cells at 30 uM by indirect affinity mass spectrometry assay	Target-based assay	1	cell-based format
CHEMBL2183041	Binding affinity to Akt1 (144 to 480) expressed in Sf9 cells assessed as fluorescence quenching by Trp fluorescence assay	Target-based assay	2	cell-based format
CHEMBL2183042	Binding affinity to Akt1 (1 to 480) expressed in Sf9 cells assessed as fluorescence quenching by Trp fluorescence assay	Target-based assay	2	cell-based format
CHEMBL2183043	Binding affinity to Akt1 (144 to 480) expressed in Sf9 cells assessed as change in melting temperature at 1.2 mM	Target-based assay	1	cell-based format
CHEMBL2183044	Binding affinity to Akt1 (1 to 480) expressed in Sf9 cells assessed as change in melting temperature at 1.2 mM	Target-based assay	1	cell-based format
CHEMBL2183045	Binding affinity to Akt1 (1 to 480) expressed in Sf9 cells assessed as change in melting temperature at 15 uM	Target-based assay	1	cell-based format
CHEMBL2183046	Binding affinity to Akt1 (144 to 480) expressed in Sf9 cells assessed as change in melting temperature at 15 uM	Target-based assay	1	cell-based format
CHEMBL2183047	Binding affinity to Akt1 (1 to 480) expressed in Sf9 cells assessed as change in melting temperature at 30 uM	Target-based assay	33	cell-based format

CHEMBL2186223	Inhibition of Akt1 (1 to 480) expressed in Sf9 cells using biotin-GRPRTSSFAEG as substrate after 20 mins by Alpha-screen assay	Target-based assay	33	cell-based format
CHEMBL2186658	Inhibition of Akt1 (1 to 480) expressed in Sf9 cells in presence of A674563	Target-based assay	1	cell-based format
CHEMBL2187087	Binding affinity to Akt1 (1 to 480) expressed in Sf9 cells by SPR analysis	Target-based assay	1	cell-based format
CHEMBL2212878	Inhibition of human recombinant AKT1 expressed in insect cells using CKRREILSRRPSYRK as substrate assessed as residual activity at 1 uM after 60 mins relative to staurosporine	Target-based assay	1	cell-based format
CHEMBL3124155	Binding affinity to Akt in human SQ20B cells after 10 to 12 hrs by pull down assay	Target-based assay	1	cell-based format
CHEMBL3390657	Inhibition of human recombinant Akt1/PKBalpha expressed insect cells using CKRREILSRRPSYRK substrate assessed as remaining enzyme activity at 1 uM after 60 mins relative to control	Target-based assay	1	cell-based format
CHEMBL3705737	Kinase Inhibition Assay: Preparation of AKT1 and AKT2 and measurement of in vitro inhibitory activity of the above-mentioned compounds against AKT1 and AKT2 kinase activity were carried out with reference to the method disclosed in Biochem. J. Vol. 385 pp 399-408 (2005). In the preparation of AKT1 and AKT2 human AKT1 and AKT2 to which a middle T antigen tag was added were expressed in Sf 9 insect cells and then AKT1 and AKT2 were prepared following affinity purification and activation by PDK1. The prepared AKT1 and AKT2 were stored at ÃƒÂ¢ 80C. until the time of measurement of inhibitory activity of the compounds. In the measurement of inhibitory activity of the compounds AKT1 or AKT2 and each compound of the present invention were preincubated at 25C. for 120 minutes in a buffer solution for reaction (15 mM Tris-HCl pH 7.5 0.01% Tween-20 2 mM DTT). As a substrate biotinylated Crosstide (bioton-KGSGSGRPRTSSFAEG) MgCl2 and ATP were added to final concentrations.	Target-based assay	64	cell-based format
CHEMBL3705788	TR-FRET Assay: Akt1 inhibitory activity of compounds of the present invention may be quantified0 employing the Akt1 TR-FRET assay as described in the following paragraphs. His-tagged human recombinant kinase full-length Akt1 expressed in insect cells was purchased form Invitrogen (part number PV 3599). As substrate for the kinase reaction the biotinylated peptide biotin-Ahx-KKLNRTLSFAEPG (C-terminus in am- ide form) was used which can be purchased e.g. from the company Biosynthan GmbH (Berlin-Buch Germany).For the assay 50 nl of a IOOfold concentrated solution of the test compound in DMSO was pipetted into a black low volume 384well microtiter plate (Greiner Bio- One Frickenhausen Germany) 2 ul of a solution of Akt1 in assay buffer [50 mM TRIS/HCI pH 7.5 5 mM MgCI2 1 mM dithiothreitol 0.02% (v/v) Triton X-100 (Sigma)] were added and the mixture was incubated for 15 min at 22C to allow pre-binding of the test compounds to the enzyme before the start of the kinase reaction.	Target-based assay	93	cell-based format
CHEMBL3706239	TR-FRET assay: Akt1 inhibitory activity of compounds of the present invention was quantified employing the Akt1 TR-FRET assay as described in the following paragraphs. His-tagged human recombinant kinase full-length Akt1 expressed in insect cells was purchased form Invitrogen (part number PV 3599). As substrate for the kinase reaction the biotinylated peptide biotin-Ahx-	Target-based assay	38	cell-based format

	KKLNRTLSFAEPG (C-terminus in amide form) was used which can be purchased e.g. from the company Biosynthan GmbH (Berlin-Buch Germany).For the assay 50 nl of a 100 fold concentrated solution of the test compound in DMSO was pipetted into a black low volume 384 well microtiter plate (Greiner Bio-One Frickenhausen Germany) 2 ul of a solution of Akt1 in assay buffer [50 mM TRIS/HCl pH 7.5 5 mM MgCl2 1 mM dithiothreitol 0.02% (v/v) Triton X-100 (Sigma)] were added and the mixture was incubated for 15 min at 22 C. to allow pre-binding of the test compounds to the enzyme before the start of the kinase reaction.			
CHEMBL3777508	Inhibition of N-terminal His6-tagged recombinant human PKBalpha expressed in baculovirus infected insect Sf9 cells assessed as remaining activity at 1 uM relative to control	Target-based assay	3	cell-based format
CHEMBL3820356	Inhibition of full length recombinant human N-terminal His6-tagged PKB-alpha expressed in baculovirus infected Sf21 cells at 1 uM by radiometric assay relative to control	Target-based assay	2	cell-based format
CHEMBL3880964	Inhibition of full length human AKT1 expressed in Sf9 cells assessed as reduction in substrate phosphorylation using biotin-ahx-ARKRERAYSFGHHA-amide substrate and [gamma-33P]ATP incubated for 40 mins by top count microplate scintillation counting method	Target-based assay	21	cell-based format
CHEMBL3888207	Akt1 TR-FRET assay: His-tagged human recombinant kinase full-length Akt1 expressed in insect cells was purchased form Invitrogen (part number PV 3599). As substrate for the kinase reaction the biotinylated peptide biotin-Ahx-KKLNRTLSFAEPG (C-terminus in amide form) was used which can be purchased e.g. from the company Biosynthan GmbH (Berlin-Buch Germany). For the assay 50 nl of a 100 fold concentrated solution of the test compound in DMSO was pipetted into a black low volume 384 well microtiter plate (Greiner Bio-One Frickenhausen Germany) 2 Î¼l of a solution of Akt1 in assay buffer [50 mM TRIS/HCl pH 7.5 5 mM MgCl2 1 mM dithiothreitol 0.02% (v/v) Triton X-100 (Sigma)] were added and the mixture was incubated for 15 min at 22 Â°C. to allow prebinding of the test compounds to the enzyme before the start of the kinase reaction. Then the kinase reaction was started by the addition of 3 Î¼l of a solution of adenosine-tri-phosphate (ATP 16.7 Î¼M=>final conc. in the 5 Î¼l assay volume is 10 Î¼M) and substrate (1.67 Î¼M=>final conc. in the 5 Î¼l assay volume is 1 Î¼M) in assay buffer and the resulting mixture was incubated for a reaction time of 60 min at 22 Â°C. The concentration of Akt1 in the assay was adjusted depending of the activity of the enzyme lot and was chosen appropriate to have the assay in the linear range typical enzyme concentrations were in the range of about 0.05 ng/Î¼l (final conc. in the 5 Î¼l assay volume). The reaction was stopped by the addition of 5 Î¼l of a solution of HTRF detection reagents (200 nM streptavidine-XL665 [Cisbio] and 1.5 nM anti-phosho-Serine antibody [Millipore cat. #35-001] and 0.75 nM LANCE Eu-W 1024 labeled anti-mouse IgG antibody [Perkin Elmer]) in an aqueous EDTA-solution (100 mM EDTA 0.1% (w/v) bovine serum albumin in 50 mM HEPES/NaOH pH 7.5). The resulting mixture was incubated 1 h at 22 Â°C. to allow the binding of the biotinylated phosphorylated peptide to the streptavidine-XL665 and the antibodies. Subsequently the amount of phosphorylated substrate was evaluated by measurement of the resonance energy transfer from the anti-mouse-IgG-Eu-Chelate to the streptavidine-XL665. Therefore the fluorescence emissions at 620 nm and 665 nm after excitation at 350 nm was measured in a HTRF reader e.g. a Rubystar (BMG Labtechnologies Offenburg	Target-based assay	8	cell-based format

	Germany) or a Viewlux (Perkin-Elmer). The ratio of the emissions at 665 nm and at 622 nm was taken as the measure for the amount of phosphorylated substrate. The data were normalised (enzyme reaction without inhibitor=0% inhibition all other assay components but no enzyme=100% inhibition). Normally test compound were tested on the same microtiter plate at 10 different concentrations in the range of 20 Î¼M to 1 nM (20 Î¼M 6.7 Î¼M 2.2 Î¼M 0.74 Î¼M 0.25 Î¼M 82 nM 27 nM 9.2 nM 3.1 nM and 1 nM dilution series prepared before the assay at the level of the 100 fold conc. stock solutions by serial 1:3 dilutions) in duplicate values for each concentration and IC50 values were calculated by a 4 parameter fit using an in-house software.			
CHEMBL4121773	Inhibition of human recombinant AKT1 expressed in insect cells incubated for 60 mins measured at apparent ATP Km level by kinase ADP-FP assay	Target-based assay	4	cell-based format
CHEMBL4178299	Inhibition of recombinant human full length N-terminal GST-tagged AKT1 expressed in baculovirus infected Sf9 cells assessed as change in enzyme activity at 1 uM in presence of 2 uM ATP relative to control	Target-based assay	2	cell-based format
CHEMBL4180876	Inhibition of recombinant human full length His6-tagged PKBa (118 to 480 residues) S473D mutant expressed in Sf21 insect cells assessed as residual activity at 1 uM after 30 mins in presence of [gamma-33P]ATP relative to control	Target-based assay	1	cell-based format
CHEMBL4181198	Inhibition of recombinant human N-terminal His6-tagged PKBalpha (118 to end residue) S473D mutant expressed in baculovirus Sf21 insect cells at 10 uM by filter binding radioactive ATP transferase assay relative to control	Target-based assay	1	cell-based format
CHEMBL4231206	Inhibition of recombinant human AKT1 expressed in insect cells using CREBtide as substrate after 60 mins by LANCE method	Target-based assay	1	cell-based format
CHEMBL4266615	Inhibition of recombinant human N-terminal His6-tagged full length PKBalpha expressed in baculovirus infected Sf21 insect cells at 1 uM relative to control	Target-based assay	3	cell-based format
CHEMBL4267274	Inhibition of recombinant human N-terminal His-tagged full length human AKT1 (1 to 480 residues) expressed in baculovirus infected Sf9 insect cells at 1 uM using FAM-labeled peptide as substrate after 10 mins by mobility shift assay relative to control	Target-based assay	6	cell-based format
CHEMBL4349768	Inhibition of recombinant human N-terminal GST/His6-tagged and C-terminal His6-tagged AKT1(V106 to A480) expressed in Sf9 insect cells at 20 nM using phosphatidylserine as substrate incubated for 60 mins in presence of [33P]-ATP by scintillation counting method	Target-based assay	1	cell-based format
CHEMBL4384269	Inhibition of PKBalpha (unknown origin) expressed in Sf9 insect cells using Biotin-SGRARTSSFAEPG as substrate after 30 mins by ELISA	Target-based assay	1	cell-based format
CHEMBL4424902	Inhibition of human full-length N-terminal His6-tagged PKBalpha expressed in baculovirus infected Sf21 insect cells using Crosstide as substrate	Target-based assay	1	cell-based format
CHEMBL4478653	Inhibition of full length active AKT1 (1 to 480 residues) (unknown origin) expressed in baculovirus infected Sf9 insect cells using biotin-GRPRTSSFAEG as substrate preincubated for 20 mins followed by substrate/PDK1/MAPKAPK2/DOPS/DOPC/PtdIns(345)P3 addition measured after 30 mins by Alphascreen assay	Target-based assay	4	cell-based format

CHEMBL4478656	Inhibition of full length unphosphorylated AKT1 (1 to 480 residues) (unknown origin) expressed in baculovirus infected Sf9 insect cells using biotin-GRPRTSSFAEG as substrate preincubated for 20 mins followed by substrate addition measured after 30 mins by Alphascreen assay	Target-based assay	21	cell-based format
CHEMBL928489	Inhibition of human Akt1 in human C33A cells by immunoprecipitation kinase assay	Target-based assay	16	cell-based format
CHEMBL939831	Inhibition of human cloned Akt1 expressed in Drosophila S2 cells by HTRF assay	Target-based assay	10	cell-based format
CHEMBL984696	Inhibition of human PKBalpha expressed in insect Sf9 cells at 10 uM	Target-based assay	2	cell-based format

Table S2. Example of application on AKT1 protein. Most recursive cell lines in ChEMBL and PubChem cell-based datapoints. For each cell line, the corresponding "Assay ChEMBL ID" and "PubChem AID" are indicated.

cell line	ChEMBL		PubChem	
	Assay ChEMBL ID [a]	datapoints [a]	AID [a]	datapoints [a]
PC3	CHEMBL3101074,CHEMBL1028096, CHEMBL2020675,CHEMBL3887079, CHEMBL3706189,CHEMBL3706188	383	1342867,1258722, 1258721,1061044, 657009	365
U87MG	CHEMBL1828261,CHEMBL1102924, CHEMBL1775878,CHEMBL1074388	87	595410,465047, 457296,616012	87
LNCaP	CHEMBL1632821,CHEMBL2188026, CHEMBL1764866	81	708170,549235, 590452,591325	99
A2780	CHEMBL2186213,CHEMBL2186215, CHEMBL2186217	45	706834,706836, 706838	45
AN3CA	CHEMBL2186214,CHEMBL2186216, CHEMBL2186218	45	706839,706835, 706837	45
C33a	CHEMBL951579,CHEMBL950115	44	344174,317080	44
BT20	CHEMBL2379605	25	744341	25
U87	CHEMBL936669,CHEMBL1176555	22	492682,319620	22
PC3M	CHEMBL1074386	15	457294	15
A549	CHEMBL2349705	13	738313	13
HT-29	CHEMBL3888334	12	1344129	12
BT474			391378	20

[a] Only assays with at least 10 associated datapoints were herein considered.
